# Demographic and Nutritional Profile of Thalassemia Carrier and Non‐Carrier Young Children in Bangladesh: A Comparative Study

**DOI:** 10.1002/fsn3.72545

**Published:** 2026-11-08

**Authors:** Sabuktagin Rahman, Farhana Rinky, Santhia Ireen, Faruk Ahmed

**Affiliations:** ^1^ Department of Public Health, Faculty of Health and Life Sciences American International University‐Bangladesh Dhaka Bangladesh; ^2^ Institute of Nutrition and Food Science University of Dhaka Dhaka Bangladesh; ^3^ Brac James P. Grant School of Public Health Brac University Dhaka Bangladesh; ^4^ Public Health Unit, School of Medicine and Dentistry Griffith University Gold Coast QLD Australia

**Keywords:** Bangladesh, child health, linear growth, micronutrient intake, nutritional status, Thalassemia carrier

## Abstract

Thalassemia is a congenital hemoglobin disorder characterized by genetic defects in hemoglobin synthesis resulting in premature breakdown of the red blood cells. Clinically, the condition ranges from the asymptomatic carrier state (thalassemia trait) to overt disease (thalassemia syndrome). Globally, 7% of the population are suffering from thalassemia carrier. Children with thalassemia are at high risk of long‐term growth and nutritional deficits. There is a scarcity of data on the association of thalassemia carrier and the socio‐demographic and nutritional status. This secondary analysis nested in a clinical trial compared the socio‐demographic, nutritional, and dietary characteristics between the thalassemia carrier (*n* = 29) and non‐carrier children (*n* = 130) aged 2–5 years in a rural setting in Bangladesh. The intake of the macro‐ and micronutrients was estimated by a seven‐day semi‐quantitative food frequency questionnaire. Z‐scores corresponding to stunting (HAZ), wasting (WHZ), and underweight (WAZ) were estimated. Welch's *t*‐test was used to compare the groups. Multivariable linear regression was used to assess the association of the carrier status with the growth outcomes. The children of the carrier groups had lower HAZ than that of non‐carrier groups; −1.84 vs. −1.38, *p* = 0.02. The carrier group consumed a lower amount of vitamin D (*p* = 0.03). Thalassemia carrier status predicted lower HAZ after adjustment with the socio‐demographics, morbidities, and dietary intake (coefficient: −0.54, *p* = 0.02), which attenuated upon inclusion of hemoglobin in the model (coefficient: −0.34, *p* = 0.14). Thalassemia carrier children had lower growth potential than the non‐thalassemic peers. Amid the pertinent explanatory factors considered, overall, the thalassemia carrier state was not associated with lower growth in young children. A larger study is required for further insights.

## Graphical abstract

The study assessed the demographic and nutritional profiles of children aged 2–5 years with and without thalassemia carrier status in Belkuchi, Sirajganj, Bangladesh. Thalassemia carrier children had significantly lower height‐for‐age z‐scores (HAZ), indicating poorer linear growth. Macronutrient intake was comparable between groups, although carrier children had lower vitamin D intake. Overall, the carrier status was not associated with lower HAZ. A larger study is required for further insights.

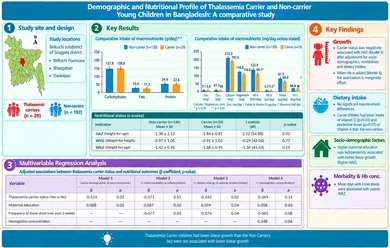

## 1 Introduction

Thalassemia is an inherited disorder caused by genetic defects that impair hemoglobin synthesis, with severity ranging from life‐threatening prenatal disease to mild anemia later in life. A key feature is expanded erythroid bone marrow activity, where increased red blood cell production often fails to produce fully functional cells, leading to abnormal hemoglobin and ineffective erythropoiesis (Rahman et al. 2023; Wendt et al. 2023). It is broadly classified into α‐thalassemia (reduced alpha‐globin production), and β‐thalassemia (reduced or absent beta‐globin). Clinically, it ranges from an asymptomatic carrier state (thalassemia trait) to overt disease, categorized as thalassemia minor (mild or no symptoms), intermedia (moderate to severe symptoms, usually not blood transfusion‐dependent), and major (Cooley's anemia), a severe form requiring regular transfusions (Galanello and Origa 2010; Hossain et al. 2025).

Thalassemia has emerged as a major global public health concern, with wide geographic variation (Musallam et al. 2023). It is prevalent across Southeast Asia, the Indian subcontinent, the Mediterranean, Central Asia, the Middle East, and West Africa—regions collectively known as the global thalassemia belt (Marengo‐Rowe 2007) that overlap with malaria‐endemic areas (Peters et al. 2012). Approximately 56,000 affected newborns are reported annually (Eleftheriou and Angastiniotis 2021), and about 7% of the global population are carriers of thalassemia traits (Mashon et al. 2025). In Bangladesh, the 2024 National Thalassemia Survey (ages 14–35 years) reported an overall carrier prevalence of 11.4%, with β‐thalassemia trait accounting for approximately 2%–3% of the population. The survey also highlighted marked regional variation, with the highest prevalence in Rangpur Division (27.7%), while Sylhet Division had the lowest rate at 4.8% (BBS 2024).

Clinical manifestations vary widely depending on genotype and disease severity (Sadiq et al. 2024). Thalassemia minor typically causes mild anemia and is often asymptomatic, though some individuals report fatigue or increased susceptibility to infections (Faizan et al. 2023; Mustafa et al. 2016). While thalassemia major poses a significant clinical burden, thalassemia carriers are important from a public health perspective due to their role in genetic transmission and potential psychosocial and nutritional vulnerabilities (Shang and Xu 2017).

Children with thalassemia carriers are at high risk of long‐term growth and nutritional deficits (Ayukarningsih et al. 2022) with growth failure affecting 25%–75% of patients (Fung 2010). Causes are multifactorial, including inadequate dietary intake, socioeconomic constraints, and limited parental literacy and awareness, which collectively reduce access to sufficient calories and micronutrients, leading to multiple micronutrient deficiencies (Correia 2018; Skordis and Kyriakou 2011). Reduced growth may also result from increased metabolic demands from chronic anemia. Gastrointestinal disturbances can further impair appetite, digestion, and nutrient absorption. Liver dysfunction secondary to iron overload (siderosis), dysglycemia (insulin resistance or deficiency), and reduced insulin‐like growth factor 1 (IGF‐1) contribute additional metabolic challenges, while oxidative stress from iron accumulation suppresses anabolism and promotes catabolism (Musallam et al. 2013; Schnedl et al. 2017). In children with thalassemia, elevated erythropoiesis increases requirements for nutrients such as folate, vitamin D, and zinc, with folate deficiency being common (Mirhosseini et al. 2012; Suh et al. 2001).

Despite evidence linking thalassemia—particularly severe forms—to malnutrition in young children (Ara et al. 2023; Ayukarningsih et al. 2022; Biswas et al. 2020; Mirhosseini et al. 2012; Soliman et al. 2023; Wang et al. 2024), research regarding the link between thalassemia carriers and malnutrition is limited.

Although Bangladesh lies within the global thalassemia belt, molecular surveys suggest that carriers of beta‐thalassemia and related hemoglobin variants form a considerable, largely undiagnosed reservoir within the population (Noor et al. 2020). Nationwide carrier‐detection studies have mapped the genetic burden of beta‐thalassemia mutations and hemoglobin E variants across the country, confirming that heterozygous carriers are common yet clinically invisible, as the condition is usually asymptomatic and identified only incidentally (Noor et al. 2020). Carrier status is nonetheless biologically consequential: the beta‐thalassemia trait is associated with altered erythropoiesis and disturbances in iron absorption and utilization that could plausibly influence growth, micronutrient status, and overall nutritional status in children (Goldberg et al. 2022; Guimaraes et al. 2015). Despite this pathway, data on the health and nutritional status of thalassemic carriers remain scarce.

Compounding this gap, no comprehensive national programme exists in Bangladesh for public education, population‐based screening, or carrier management, and premarital and antenatal screening remain voluntary, fragmented, and constrained by religious, cultural, and logistical barriers (Suresh et al. 2023). Carriers are therefore neither systematically identified nor followed for their nutritional trajectories, and nutrition programmes, including iron and micronutrient supplementation, continue to target the general population without disaggregating carriers from non‐carriers, risking underserved deficient non‐carriers or inappropriately iron‐loaded carriers.

A study comparing demographic and nutritional profiles of beta‐thalassemia carriers and non‐carriers among Bangladeshi children is therefore scientifically warranted and practically urgent. Such evidence would clarify whether carrier status independently predicts undernutrition or micronutrient imbalance and inform risk‐stratified screening and supplementation guidelines within existing child health platforms.

## 2 Materials and Methods

### 2.1 Study Site and Design

The cross‐sectional study was conducted to compare the demographic and nutritional status of the thalassemic carrier and non‐carrier children aged 2–5 years in three unions (Smallest Administrative Area in Bangladesh)—Belkuchi Pourosava, Bhangabari, and Daulatpur—within Belkuchi subdistrict in Sirajganj district in Bangladesh. The details of the study site are provided elsewhere (Rahman et al. 2019).

### 2.2 Study Population, Sample Size and Sampling

This secondary analysis used data from a randomized controlled trial evaluating low‐dose iron micronutrient powder (low‐iron MNP) in children under five in a high groundwater iron setting (Rahman et al. 2019). Among 222 screened children, 29 were identified as thalassemia carriers (including Hemoglobin E Trait, Beta Thalassemia Trait, Hemoglobin E Disease, and Hemoglobin D Punjab) and 193 were non‐carriers. There is a heterogeneity among the 29 carriers. Except for the hemoglobin E disease (1 case), all other three Trait conditions present with mild disease, mostly asymptomatic, diagnosed incidentally and life expectancy is consistent with normal (Jawed et al. 2024; Yogalakshmi et al. 2024). Hence, we considered these different disorders as a single homogenous group (i.e., thalassemia carriers) emphasizing on roughly similar functional outcomes. The large difference in group size between the thalassemia carriers and non‐carriers undermines the power to detect the group differences. We estimated that at 5% alpha, a modest effect size for the mean group difference of 0.25, with group size ratio of 4.5 to 1, the estimated power was 80.6%, which is optimum in epidemiological observational studies (StatsCalculators 2026). This entails, for the thalassemia carrier group size n1 = 29; the non‐carrier group size is roughly n2 = 130. The non‐carrier samples (n2 = 130) were selected by simple random sampling by an online random number generator (Random.org 2026) from 193 non‐carriers and considered for analysis.

### 2.3 Measurement of Key Variables

Inclusion of variables in the analysis bears association with child nutritional outcomes (Ndovie et al. 2025; Schlaudecker et al. 2011; van Neerven 2025). Socio‐demographic characteristics included mother's education, household head's occupation, number of household members, type of households, toilet types, household expenses on foods, and household food insecurity scores. Child level variables included age, sex, number of days with exclusive breastfeeding, number of days suffered from loose stool, fever, common cold over the preceding 2 weeks, and the inflammation biomarker, Alpha‐1 acid glycoprotein (AGP). Nutritional status indicators considered were—height‐for‐age Z score (HAZ), weight‐for‐height Z score (WHZ), and weight‐for‐age Z score (WAZ). Dietary intake covered both macronutrients (protein, animal‐source protein, carbohydrates, and fats) and micronutrients (iron, zinc, calcium, magnesium, vitamins A, D, and C, and folates).

### 2.4 Anthropometric Indices Measurement

Anthropometric measurements were conducted using standardized procedures. The height of young children was measured using locally constructed wooden stadiometers, and body weight was measured with a Tanita UM070 digital scale. All measurements followed World Health Organization (WHO) guidelines as described in the Food and Nutrition Technical Assistance anthropometry manual (Cashin and Oot 2018). Each measurement was taken at least twice, and when the difference between the two readings exceeded the predetermined tolerance limits (0.1 kg for weight and 0.5 cm for height), a third measurement was obtained. Nutritional status among young children was assessed using three anthropometric indices using the WHO guidelines: height‐for‐age, weight‐for‐height, and weight‐for‐age z‐scores (WHO 2006).

### 2.5 Dietary Intake Measures

Dietary intake, including energy and key macro‐ and micronutrients, was assessed using a seven‐day semi‐quantitative food frequency questionnaire (7‐day SQFFQ) (Rahman et al. 2021). This validated tool captures the actual frequency of consumption over the preceding week. It included 53 commonly consumed foods representative of the traditional Bangladeshi diet. Foods represented major groups such as cereals, legumes, vegetables, fruits, fish, meat, eggs, organ meats, and processed foods. Portion sizes were estimated using a food album with standardized photographs of serving size. Detailed methodology is described elsewhere (Rahman et al. 2021). Nutrient intakes were calculated using an updated food composition table for Bangladeshi foods (Shaheen et al. 2013). For nutrients not available in this table, values were obtained from the USDA nutrient database (USDA 2026). Edible portion coefficients and yield factors (Shaheen et al. 2013) were applied to convert cooked foods into raw edible weights, and nutrient values were calculated per 100 g of raw food. For uncooked and ready‐to‐eat processed foods, nutrient values were obtained directly from food composition tables and product nutrition labels when available.

### 2.6 Household Food Expenditure Assessment

Weekly household food expenditure was estimated by assessing spending on major food items consumed at the households' level, such as rice, flour, oil, sugar, salt, fish, meats, vegetables, and groceries. Respondents were asked about the frequency of purchases and the average amount spent per purchase over the preceding week. Total weekly food expenditure was calculated by multiplying the amount spent per purchase by the frequency of purchases for all food items.

### 2.7 Wealth Index Measurement

The household wealth index was constructed using key socio‐economic indicators, including maternal education, occupation of the household head, family size, number of dwelling rooms, housing type, sanitation facilities, possession of household assets, land ownership, and household food expenditure. Principal component analysis (PCA) was applied to derive weights for each asset variable based on the first principal component, and a composite wealth score was calculated for each household (Vyas and Kumaranayake 2006).

### 2.8 Household Food Insecurity Score

Household food insecurity was assessed using the Household Food Insecurity Access Scale (HFIAS), developed by the Food and Nutrition Technical Assistance Project (FANTA) (Coates et al. 2007). The HFIAS is a validated nine‐item questionnaire that evaluates household access to food over the preceding 4 weeks (30 days). The tool captures three domains of food insecurity: anxiety and uncertainty about food supply, insufficient quality of food, and insufficient food intake. For each occurrence question, respondents reported the frequency as rarely (1–2 times), sometimes (3–10 times), or often (more than 10 times). Responses were scored from 0 to 3, yielding a total HFIAS score ranging from 0 to 27, with higher scores indicating greater food insecurity (Coates et al. 2007).

### 2.9 Blood Sample Collection, Processing, Screening of Thalassemia and Assessment of α‐1 Acid Glycoprotein (AGP)

At the time of the survey, venous blood samples were collected from children by venipuncture of the ante‐cubital vein under appropriate aseptic conditions. An aliquot of whole blood sample was taken into EDTA tubes for the measurement of hemoglobin concentration and hemoglobinopathies. Hemoglobin levels were measured immediately using a portable photometer (HemoCue 301, Ängelholm, Sweden). The thalassemia carrier state was determined using capillary zone electrophoresis of hemoglobin at pH 9.4 with the Capillary 2 system (Sebia, Évry, France). α‐1—acid glycoprotein (AGP) was used as a biomarker of inflammation reflecting subacute or chronic inflammatory status over a period of a week or longer (Thurnham et al. 2015). AGP concentration was measured using a particle‐enhanced immunoturbidimetric assay on an automated, software‐controlled clinical chemistry analyzer (Cobas c311, Roche Diagnostics GMBH, Mannheim 68,305 Germany) with commercial assay kits. Further methodological details are provided elsewhere (Rahman et al. 2019).

### 2.10 Assessment of Child Morbidity

The occurrences of common childhood illnesses—including cough, fever, and loose stool—during the preceding 2 weeks were assessed based on caregiver's recall using standardized questions adopted from the Bangladesh Demographic and Health Surveys (BDHS 2022).

### 2.11 Statistical Analysis

Socio‐demographic characteristics of household and child‐specific variables were summarized as mean ± standard deviation (SD) for continuous variables and as frequencies and percentages [*n* (%)] for categorical and binary variables. Differences in characteristics between thalassemia carriers and the non‐carriers were assessed using the chi‐square test for categorical variables, with the chi‐square statistic and corresponding degrees of freedom reported. For continuous variables, Welch's *t*‐test was applied to account for unequal variances and substantially unequal group sizes, thereby protecting from inflation of the risk of Type I error (Delacre et al. 2017). The Welch's *t* statistic and Satterthwaite‐adjusted degrees of freedom were reported. For both the chi‐square and the Welch's *t*‐test, a *p*‐value ≤ 0.05 was considered statistically significant. The adjustment of alpha for assessing the difference in the intake of different nutrients between thalassemia carrier and non‐carrier groups was not considered. We considered testing for each nutrient as an exploratory individual independent sample Welch's *t*‐test where a joint hypothesis was not evaluated. Further, adjustment of alpha might inflate Type 2 errors (false negatives) (Perneger 1998), where it is biologically plausible that the difference is likely to exist in nutrients intake between the groups. Comparative intake of macro‐ and micronutrients were illustrated using bar diagram with 95% confidence interval (CI) error bars. Multivariable linear regression analyses were conducted to examine the association between thalassemia carrier status and anthropometric z‐scores for stunting, wasting and underweight. Relevant demographic and socio‐economic factors (Barik et al. 2026; Na et al. 2020; Nyirenda et al. 2025; Rahayuwati et al. 2023; Semba et al. 2008; Vongsakit et al. 2025), morbidity indicators (Arini et al. 2020; Weisz et al. 2011) and dietary intake variables (Headey et al. 2018; Mahfouz et al. 2022; Zaharia et al. 2021) were included as covariates in the model in a stepwise manner from the parsimonious (model 1) to the full model (model 4). Model validity was assessed by *F* statistic (df) along with *p*‐values. Overall model fit was assessed by *R* ^2^ (Adjusted *R* ^2^). Mean Variance Inflation Factor (VIF) was estimated to assess the multicollinearity. Associations were reported as regression coefficient, *t*‐statistic and the *p*‐values. Missing data points were found for the participants who did not consume particular foods from the list of the SQFFQ. The missing data were replaced by “zero” to estimate the average intake of foods without the compromise of sample numbers. All analyses were performed using STATA version 17.0 and ggplot2 commands in R‐Studio.

## 3 Ethical Consideration and Approval

The study was nested within a randomized controlled trial. The trial was approved by the Research Ethical Committee of the Faculty of Biological Science, Dhaka University, Bangladesh (Ref# 46/Biol. Scs./2017–2018) and the Griffith University Human Research Ethics Committee, Australia (Ref# 2017/467). The study was compliant with the Helsinki Ethical Standards.

## 4 Results

The study analyzed data from 159 children, of whom 130 were thalassemia non‐carriers and 29 were carriers. Among the carriers, the distribution of types was as follows: Hemoglobin E trait (82.7%), Beta Thalassemia Trait (10.3%), Hemoglobin E Disease (3.45%) and Hemoglobin D Punjab (3.45%). The socio‐demographic characteristics of the participants by carrier status are presented in Table 1.

**TABLE 1:** Socio‐demographic characteristics of the participants stratified by thalassemia carrier status.

Measures	Thalassemia non‐carrier (*n* = 130)	Thalassemia carrier (*n* = 29)	Statistical report	*p*
*n* (%); mean ± SD				
Mother's education (in years)	5.50 ± 3.31	5.82 ± 2.76	*T* statistic: −0.54 Satterthwaite's degrees of freedom: 47.77	0.59
Occupation of household head				
Business	20 (15.38)	5 (17.40)	*χ* ^*2*^: 4.92 Degrees of freedom: 7	0.67
Factory worker	44 (33.85)	9 (31.03)		
Service	9 (6.92)	1 (3.45)		
Skilled labor	7 (5.38)	3 (10.34)		
Unskilled labor	21 (16.15)	3 (10.34)		
Farmers	14 (10.77)	4 (13.79)		
Housewife	9 (6.92)	4 (13.79)		
Others	6 (4.63)	0 (0.0)		
Number of family members	5.26 ± 2.49	4.83 ± 1.23	*T* statistic: 1.38 Satterthwaite's degrees of freedom: 86.94	0.17
Types of households				
Predominantly earthen^b^	0 (0.0)	1 (3.45)	*χ* ^*2*^: 5.00 Degrees of freedom: 3	0.17
Earth‐CI sheet^c^	94 (72.31)	19 (65.52)		
Bricks‐CI sheet^d^	35 (26.92)	9 (31.03)		
All bricks^e^	1 (0.77)	0 (0.00)		
Types of toilet facility				
Flush to septic tank^f^	16 (12.31)	1 (3.45)	*χ* ^*2*^: 6.58 Degrees of freedom: 3	0.08
Pit latrine with slab	96 (73.85)	20 (68.97)		
Pit latrine without slab	9 (6.92)	6 (20.69)		
Others	9 (6.92)	2 (6.90)		
Household asset score	0.83 ± 0.11	0.82 ± 0.09	*T* statistic: 0.19 Satterthwaite's degrees of freedom: 46.53	0.84
Household food insecurity score	3.12 ± 4.14	5.03 ± 5.90	*T* statistic: −1.66 Satterthwaite's degrees of freedom: 34.38	0.10
Weekly household expenses on food (BDT)	1785.27 ± 894.92	1473.21 ± 548.02	*T* statistic: 2.43 Satterthwaite's degrees of freedom: 66.14	**0.02** ^a^

^a^ Statistically significant at *p* < =0.05.

^b^ Floor and walls made of earth.

^c^ Floor and walls are made of earth and corrugated iron (CI) sheets respectively.

^d^ Floor and the walls are made of bricks and corrugated iron sheets respectively.

^e^ Made of bricks.

^f^ A reservoir of human excreta connected with the toilet.

The mean number of years of maternal education was 5.50 years for non‐carrier children and 5.82 years for carrier children. In both groups, the majority of fathers were factory workers (31%–33.8%). The average household size was 4.83 among carriers and 5.26 among non‐carriers. The predominant types of housing were “Earth‐CI sheet”, accounting for 72.3% of non‐carrier households and 65.5% of carrier households. Pit latrines with slabs were the most common sanitation facilities, used by 73.8% of non‐carrier households and 69% of carrier households. The average household asset score was similar between non‐carriers (0.83) and carriers (0.82).

The weekly household expenditure on food was BDT 1785.3 for non‐carrier households and BDT 1473.2 for carrier households, and this difference was statistically significant (t statistic: 2.43; Satterthwaite's degrees of freedom: 66.141; *p* = 0.02). The household food insecurity score was higher among thalassemia carrier children (5.03) than among non‐carrier children (3.12); however, this difference was not statistically significant (t statistic: −1.66; Satterthwaite's degrees of freedom: 34.38; *p* = 0.10).

Table 2 presents characteristics of children by thalassemia carrier status. The mean age of thalassemia carriers and non‐carrier children was 41.14 months and 39.67 months, respectively. The mean energy intake was 919.47 kcal/day among carriers and 931.77 kcal/day among non‐carrier children. The mean intake of animal‐source protein was 9.32 g/day among carrier children and 11.27 g/day among non‐carrier children. In terms of morbidity, thalassemia non‐carrier children experienced a significantly higher average number of days with loose stools compared with carrier children (*t* statistic: 2.39; Satterthwaite's degrees of freedom: 145.65; *p* = 0.02). The mean duration of fever was 1.16 days among non‐carrier children, compared with 0.97 days among carrier children. Similarly, the mean duration of the common cold was 3.54 days among non‐carriers and 3.48 days among carriers. However, the differences were not statistically significant for fever (*t* = 0.53; Satterthwaite's degrees of freedom = 44.16; *p* = 0.60) and common cold (*t* = 0.06; Satterthwaite's degrees of freedom = 38.91; *p* = 0.95). The mean hemoglobin concentrations were 12.36 ± 0.72 and 11.60 ± 0.59 g/dL in the non‐carriers and the carriers respectively (*t* = 6.01; Satterthwaite's degrees of freedom = 49.35; *p* < 0.0001).

**TABLE 2:** Characteristics of children by thalassemia carrier status.

Measures	Thalassemia non‐carrier (*n* = 130)	Thalassemia carrier (*n* = 29)	Statistical report	*p*
*n* (%); mean ± SD				
Age (in months)	39.67 ± 8.49	41.14 ± 9.99	*T* statistic: −0.73 Satterthwaite's degrees of freedom: 37.52	0.46
Sex				
Male	70 (53.85)	16 (55.17)	*χ* ^2^: 0.02 Degrees of freedom: 1	0.89
Female	60 (46.15)	13 (44.83)		
Number of days of EBF	39.47 ± 79.80	34.69 ± 66.20	*T* statistic: 0.34 Satterthwaite's degrees of freedom: 48.16	0.73
Energy intake (kcal/day)	931.77 ± 372.22	919.47 ± 317.99	*T* statistic: 0.18 Satterthwaite's degrees of freedom: 46.78	0.85
Animal source protein intake (g/day)	11.27 ± 9.05	9.32 ± 8.79	*T* statistic: 1.07 Satterthwaite's degrees of freedom: 42.30	0.28
Number of days suffered from loose stools	0.68 ± 2.83	0.07 ± 0.37	*T* statistic: 2.39 Satterthwaite's degrees of freedom: 145.65	**0.02** ^a^
Number of days suffered from fever	1.16 ± 1.94	0.97 ± 1.78	*T* statistic: 0.53 Satterthwaite's degrees of freedom: 44.15	0.60
Number of days suffered from common cold	3.54 ± 4.08	3.48 ± 4.51	*T* statistic: 0.06 Satterthwaite's degrees of freedom: 38.90	0.95
Mean hemoglobin (g/dL)	12.36 ± 0.72	11.60 ± 0.59	*T* statistic: 6.01 Satterthwaite's degrees of freedom: 49.35	**0.0001** ^a^
AGP level	76.15 ± 29.43	71.52 ± 21.21	*T* statistic: 0.98 Satterthwaite's degrees of freedom: 55.01	0.32

*Note:* Bold values indicate *p* < =0.05.

Abbreviations: AGP, Alpha (1)‐acid glycoprotein; EBF, Exclusive Breastfeeding.

^a^ Statistical significance at *p* < =0.05.

The nutritional status of the children by thalassemia carrier status is depicted in Table 3. The mean height‐for‐age z‐score (HAZ) was significantly lower among carrier children (−1.84 ± 0.81) compared with non‐carriers (−1.38 ± 1.12; *t* = 2.52; Satterthwaite's degrees of freedom = 54.88; *p* = 0.02). The mean weight‐for‐age z‐score (WAZ) was −1.68 ± 0.95 among carriers and −1.42 ± 0.95 among non‐carriers, while the mean weight‐for‐height z‐score (WHZ) was −0.91 ± 1.03 among carriers and −0.97 ± 1.05 among non‐carriers. However, these differences were not statistically significant.

**TABLE 3:** Nutritional status of children by thalassemia carrier status.

Nutritional status	Thalassemia non‐carrier (*n* = 130)	Thalassemia carrier (*n* = 29)	Statistical report	*p*
	Mean ± SD			
HAZ	−1.38 ± 1.12	−1.84 ± 0.81	*T* statistic: 2.52; Satterthwaite's degrees of freedom: 54.88	**0.02** ^a^
WHZ	−0.97 ± 1.05	−0.91 ± 1.03	*T* statistic: −0.29; Satterthwaite's degrees of freedom: 42.04	0.77
WAZ	−1.42 ± 0.95	−1.68 ± 0.95	*T* statistic: −1.34; Satterthwaite's degrees of freedom: 41.62	0.19

*Note:* Bold values indicate  *p* < =0.05.

Abbreviations: HAZ, Height‐for‐Age Z‐score; WAZ, Weight‐for‐Age Z‐score; WHZ, Weight‐for‐Height Z‐score.

^a^ Statistical significance at *p* < =0.05.

Figure 1 depicts the comparative intake of the key macronutrients—carbohydrates, fats and proteins, between the non‐carrier and carrier children. Intake of carbohydrates in the non‐carrier and carrier children was 137.8 and 139.8 g/day respectively (*t* = −0.175, *p* = 0.86). Fat intake was 13.4 g/day in non‐carriers and 11.3 g/day in carriers, while protein intake was 24.4 g/day and 22.6 g/day, respectively. However, these differences were not statistically significant (*p* = 0.27 for fat and *p* = 0.41 for protein).

**FIGURE 1:**
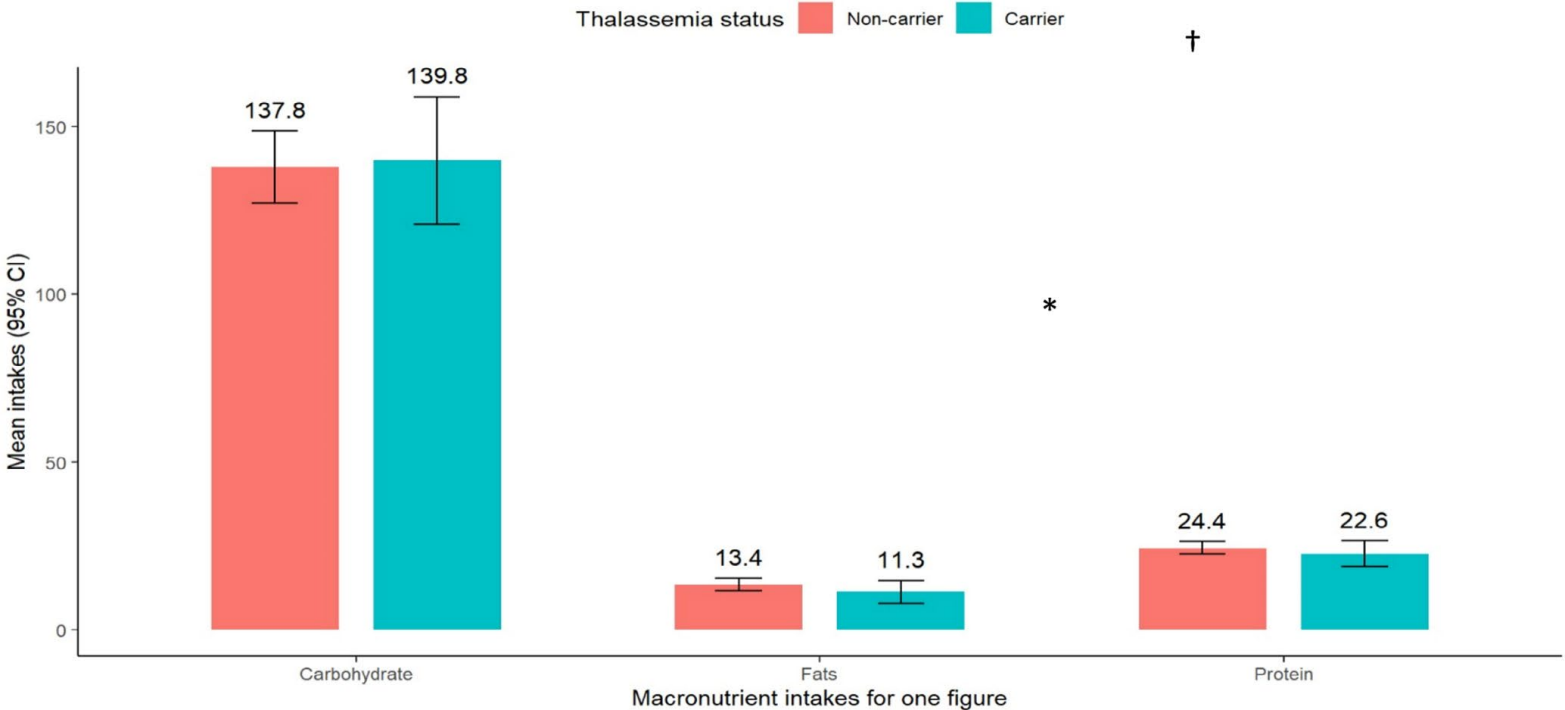
Comparative intake (g/day) of the key macronutrients in the non‐carrier and carrier children. ^†^Welch *t*‐tests were performed to assess the group difference; *No significant differences between the groups.

Figure 2 presents the comparative intake of the key micronutrients—iron, zinc, calcium, magnesium, vitamin A, vitamin D, folates, and vitamin C between the non‐carrier and carrier children. The intake of iron among non‐carrier and carrier children was 3.1 and 3.0 mg/day, respectively (*p* = 0.72). Calcium intake was 212.3 and 192.6 mg/day among non‐carriers and carrier children, respectively (*p* = 0.66). Magnesium intake was 119.8 mg/day in non‐carrier children and 116.6 mg/day in carrier children (*p* = 0.71). Zinc intake was 4.1 mg/day among non‐carrier children and 3.8 mg/day among carrier children (*p* = 0.40). Vitamin A intake was apparently higher among non‐carrier children (122.9 RAE/day) compared with carrier children (90.7 RAE/day), with marginal significance at the 7% level (*p* = 0.07). Vitamin D intake was significantly higher among non‐carrier children (0.3 mcg/day) compared with carrier children (0.2 mcg/day) (*p* = 0.03). Folate intake was 67.5 mcg/day among non‐carrier and 60.0 mcg/day among carrier children; however, the difference was not statistically significant (*p* = 0.17).

**FIGURE 2:**
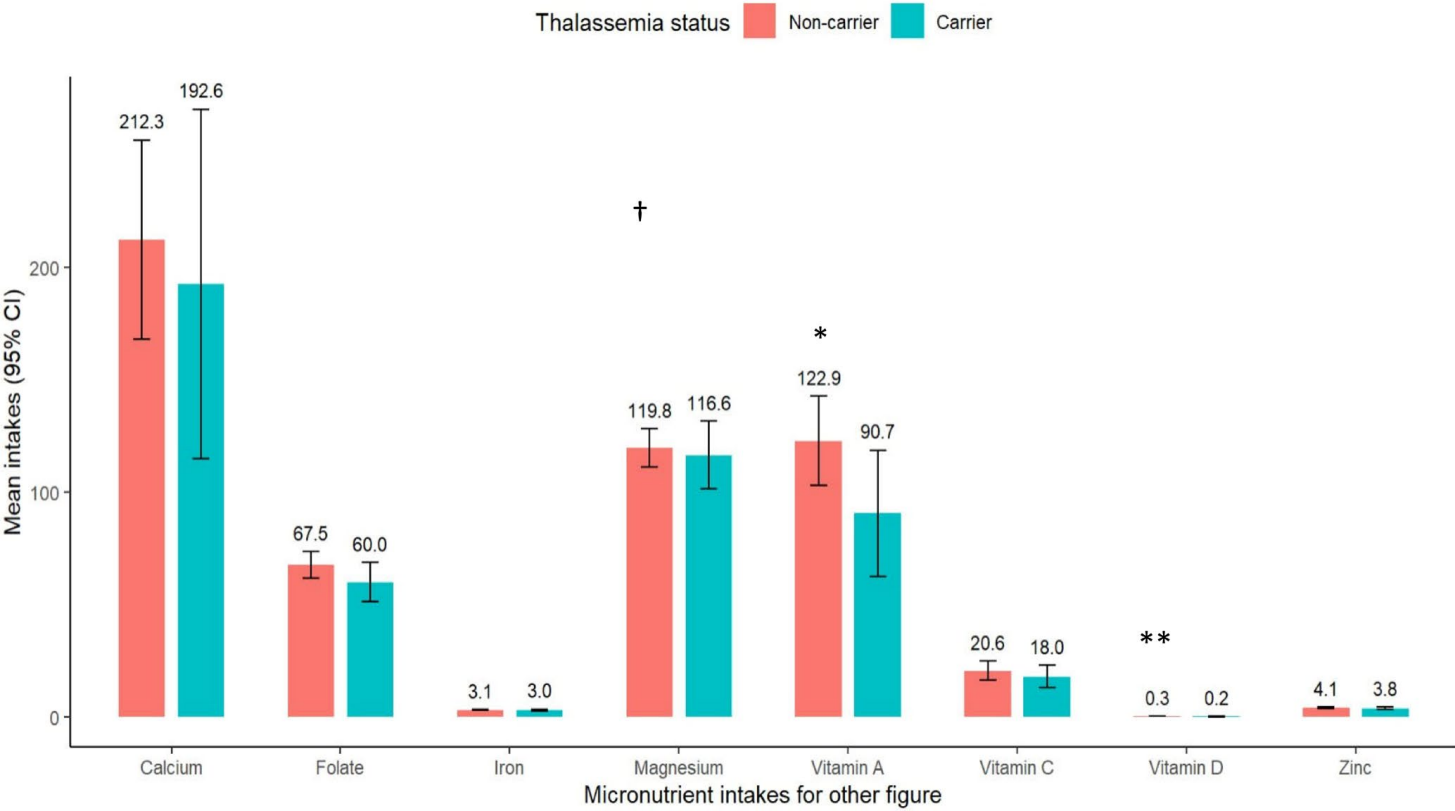
Comparative intake of the key micronutrients in the non‐carrier and carrier children. Calcium, magnesium, vitamin C, iron, and zinc are consumed in mg/day. Folate and Vitamin D are consumed in mcg/day. Vitamin A is consumed in Retinol Activity Equivalents (RAE/day). *Difference in the intake of vitamin A is marginally significant at 7% (*p* = 0.07). **Difference in the intake of vitamin D is significant (*p* = 0.03). ^†^Welch *t*‐tests were performed to assess the group differences.

Multivariable linear regression was conducted to examine the associations between thalassemia carrier status and height‐for‐age Z score (HAZ) in children, each adding a conceptually distinct block of covariates, through stepwise modeling. The parsimonious model (model 1), adjusted for demographic and socio‐economic status (child sex, household asset score, weekly spending on food, number of years of maternal education, and household food insecurity score). Model 2 additionally adjusted for child morbidity indicators such as, frequencies of loose stools, fever and common cold over the preceding 2 weeks and subclinical inflammation (alpha‐1‐acetylated glycoprotein). Model 3 additionally included dietary energy and animal‐source protein intake, while Model 4, the final model, added hemoglobin concentration (Table 4). We assessed diagnostics of regression—residual behavior, influential/high‐leverage observations with the Model 4. The diagnostics, for the final HAZ model (Model 4, *N* = 159) showed no major violation of regression assumptions. Residuals center near zero across fitted values, however it showed mild non‐normality (Shapiro–Wilk *W* = 0.982, *p* = 0.035), consistent with a slight right skew. No observation was highly influential: Cook's distances of all covariates were well below 1. Residual diagnostics for the final WAZ model (Model 4, *N* = 159) showed residuals that approximate normality (Shapiro–Wilk *W* = 0.986, *p* = 0.115) and scatter evenly around zero across fitted values. No observation was highly influential: the largest Cook's distance (0.073) was well below 1 (Results not shown). Thalassemia carrier status was significantly and negatively associated with HAZ in the Parsimonious model and models 2 and 3, indicating that carrier children had lower HAZ scores than non‐carriers after adjusting for demographic, socio‐economic, morbidity, and dietary factors (*β* were −0.52, −0.57, −0.54 respectively, *p* < 0.05). In the final model, after additionally including hemoglobin concentration, the association diminished and lost statistical significance (*β* = −0.35, *p* = 0.14), suggesting that hemoglobin status may partly explain the relationship between thalassemia carrier status and children's linear growth. Maternal education was positively associated with HAZ across all four models (*p* < 0.05). Hemoglobin concentration was positively associated with HAZ in the final model (*β* = 0.248, *p* = 0.04) complementing its independent effect, influencing the association of thalassemia carrier and HAZ. All four models were valid as the *F*‐statistic were significant *p* < 0.05. The model fit was modest but improved progressively, with Adj‐*R* ^2^ rising from 0.053 (Model 1/Parsimonious) to 0.091 (Model 4). Mean variance inflation factors (VIF) remained low across all models (1.11 to 1.40), suggesting multicollinearity was not a concern in any model.

**TABLE 4:** Multivariable linear regression models of the association between thalassemia carrier status and height‐for‐age Z‐score (HAZ) among young children in Bangladesh (*N* = 159).

Variable	Model 1			Model 2			Model 3			Model 4		
	*β*	95% CI	*p*	*β*	95% CI	*p*	*β*	95% CI	*p*	*β*	95% CI	*p*
Thalassemia carrier status (Yes vs. No)	−0.524*	−0.964, −0.084	0.02	−0.571*	−1.009, −0.134	0.01	−0.542*	−0.982, −0.102	0.02	−0.349	−0.825, 0.126	0.14
Household asset score	0.882	−0.790, 2.555	0.29	0.705	−0.968, 2.378	0.40	0.623	−1.065, 2.310	0.46	0.857	−0.829, 2.544	0.31
Household spending on food	0.000	−0.000, 0.000	0.82	0.000	−0.000, 0.000	0.30	0.000	−0.000, 0.000	0.26	0.000	−0.000, 0.000	0.25
Child sex (Female vs. Male)	0.143	−0.193, 0.479	0.40	0.136	−0.215, 0.488	0.44	0.196	−0.165, 0.557	0.28	0.186	−0.171, 0.544	0.30
Maternal education	0.068*	0.012, 0.123	0.02	0.067*	0.012, 0.123	0.02	0.059*	0.002, 0.116	0.04	0.058*	0.002, 0.115	0.04
Household food insecurity access scale score	0.032	−0.007, 0.071	0.11	0.033	−0.007, 0.072	0.10	0.033	−0.006, 0.073	0.10	0.037	−0.003, 0.076	0.06
Frequency of loose stool over past 2 weeks	—	—	—	−0.077*	−0.150, −0.004	0.03	−0.074*	−0.146, −0.001	0.04	−0.065	−0.137, 0.008	0.08
Frequency of fever over past 2 weeks	—	—	—	0.011	−0.113, 0.134	0.86	0.011	−0.113, 0.135	0.86	0.009	−0.113, 0.132	0.88
Frequency of cold over past 2 weeks	—	—	—	0.030	−0.023, 0.083	0.27	0.022	−0.032, 0.076	0.41	0.019	−0.035, 0.073	0.48
Alpha‐1‐acid glycoprotein, AGP (inflammation marker)	—	—	—	−0.006	−0.013, 0.001	0.07	−0.006	−0.013, 0.001	0.07	−0.005	−0.012, 0.003	0.20
Dietary energy intake	—	—	—	—	—	—	−0.000	−0.001, 0.000	0.21	−0.000	−0.001, 0.000	0.18
Animal‐source protein intake	—	—	—	—	—	—	0.014	−0.008, 0.035	0.21	0.012	−0.010, 0.033	0.28
Hemoglobin concentration	—	—	—	—	—	—	—	—	—	0.248*	0.002, 0.494	0.04
Constant	−2.693**	−4.069, −1.316	< 0.001	−2.283**	−3.733, −0.833	0.002	−2.046**	−3.550, −0.542	0.008	−5.397**	−9.044, −1.749	0.004
*N* (observations)	159			159			159			159		
*F* statistic (df)	*F*(6, 152) = 2.47			*F*(10, 148) = 2.21			*F*(12, 146) = 2.03			*F*(13, 145) = 2.22		
Prob > *F*	0.03			0.02			0.02			0.01		
Adjusted *R* ^2^	0.0528			0.0713			0.0725			0.0909		
Mean VIF (multicollinearity)	1.11			1.36			1.38			1.40		

*Note:* Model 1—Parsimonious (socio‐demographic & socio‐economic) | Model 2—+ child morbidity & inflammation | Model 3—+ dietary energy & animal‐protein intake | Model 4—Final (+ hemoglobin concentration). Outcome variable: height‐for‐age Z‐score (HAZ). Estimates are unstandardized OLS regression coefficients (*β*) with two‐tailed *p*‐values; * *p* < 0.05, **p < 0.01. Reference categories: Thalassemia carrier status = No; Child sex = Male. *N* = 159 for all models. Model 1 (parsimonious): Thalassemia carrier status, household asset score, household spending on food, child sex, maternal education, household food insecurity access scale score. Model 2: Model 1 + three child morbidity indicators (frequencies of loose stool, fever and cold over the past 2 weeks) + AGP (alpha‐1‐acid glycoprotein, a marker of subclinical inflammation). Model 3: Model 2 + dietary energy intake and animal‐source protein intake. Model 4 (final): Model 3 + hemoglobin concentration. Mean VIF = mean variance inflation factor across all predictors in the model.

Abbreviation: 95% CI = 95% confidence interval.

Further, multivariable linear regression was conducted to examine the associations between thalassemia carrier status and weight‐for‐age Z score (WAZ) in children (Table 5). The analysis was stepwise with four models similar to the above stated approach, except the dependent variable was WAZ. Unlike its association with HAZ, thalassemia carrier status was not significantly associated with WAZ in any model (β ranging from −0.231 to −0.037; *p* > 0.05), suggesting carrier status does not independently predict weight‐for‐age after adjusting for the confounders. Household spending on food was consistently and positively associated with WAZ across all four models, more strongly with the models 2–4 (*p* ≤ 0.003, 0.002 and 0.002 respectively). Number of years of maternal education was significant positively associated with WAZ in models 1–2 (*p* < 0.05) and in borderline positively associated in models 3–4 (*p*‐values 0.09–0.10). Child morbidity—loose stool frequency over preceding 2 weeks was significant negatively associated with WAZ in models 2 through 4 (*p*‐values 0.004, 0.004 and 0.01 respectively). In the final model, hemoglobin concentration was significant positively associated with WAZ (*β* = 0.268, *p* = 0.02), however, thalassemia carrier status remained non‐significant. Except the parsimonious model, models 2–4 were valid (*F* statistic significant at *p ≤* 0.05). The model fit was modest but improved progressively, with adj‐*R* ^2^ increased from 0.036 (Model 1/parsimonious) to 0.1 (Model 4). Mean variance inflation factors (VIF) remained low, and ranged between 1.11 and 1.40 across all models ruling out multicollinearity of any concern.

**TABLE 5:** Multivariable linear regression models of the association between thalassemia carrier status and weight‐for‐age Z‐score (WAZ) among young children in Bangladesh (*N* = 159).

Variable	Model 1			Model 2			Model 3			Model 4		
	*β*	95% CI	*p*	*β*	95% CI	*p*	*β*	95% CI	*p*	*β*	95% CI	*p*
Thalassemia carrier status (Yes vs. No)	−0.231	−0.622, 0.159	0.24	−0.272	−0.656, 0.113	0.16	−0.246	−0.633, 0.141	0.21	−0.037	−0.453, 0.378	0.85
Household asset score	−0.414	−1.898, 1.070	0.58	−0.615	−2.084, 0.855	0.41	−0.745	−2.229, 0.739	0.32	−0.491	−1.965, 0.982	0.51
Household spending on food	0.0002*	0.000, 0.000	0.04	0.0003**	0.000, 0.000	0.003	0.0003**	0.000, 0.000	0.002	0.0003**	0.000, 0.000	0.002
Child sex (Female vs. Male)	−0.094	−0.392, 0.204	0.53	−0.132	−0.442, 0.177	0.39	−0.096	−0.414, 0.221	0.55	−0.106	−0.418, 0.206	0.50
Maternal education	0.053*	0.004, 0.102	0.03	0.051*	0.002, 0.099	0.04	0.043	−0.007, 0.093	0.09	0.042	−0.008, 0.091	0.10
Household food insecurity access scale score	0.005	−0.030, 0.040	0.79	0.006	−0.029, 0.041	0.74	0.008	−0.027, 0.043	0.66	0.011	−0.023, 0.046	0.51
Frequency of loose stool over past 2 weeks	—	—	—	−0.095**	−0.158, −0.031	0.004	−0.094**	−0.158, −0.030	0.004	−0.084*	−0.148, −0.021	0.01
Frequency of fever over past 2 weeks	—	—	—	−0.019	−0.128, 0.089	0.72	−0.021	−0.130, 0.087	0.69	−0.023	−0.130, 0.084	0.66
Frequency of cold over past 2 weeks	—	—	—	0.044	−0.003, 0.091	0.06	0.041	−0.006, 0.089	0.09	0.038	−0.009, 0.085	0.11
Alpha‐1‐acid glycoprotein, AGP (inflammation marker)	—	—	—	−0.0025	−0.009, 0.004	0.41	−0.0023	−0.008, 0.004	0.45	−0.0004	−0.007, 0.006	0.89
Dietary energy intake	—	—	—	—	—	—	−0.00004	−0.000, 0.000	0.84	−0.00007	−0.001, 0.000	0.76
Animal‐source protein intake	—	—	—	—	—	—	0.012	−0.007, 0.031	0.20	0.010	−0.009, 0.029	0.28
Hemoglobin concentration	—	—	—	—	—	—	—	—	—	0.268*	0.053, 0.483	0.02
Constant	−1.674**	−2.895, −0.452	0.008	−1.550*	−2.823, −0.277	0.02	−1.537*	−2.860, −0.214	0.02	−5.165**	−8.351, −1.979	0.002
*N* (observations)	159			159			159			159		
*F* statistic (df)	*F*(6, 152) = 1.99			*F*(10, 148) = 2.27			*F*(12, 146) = 2.04			*F*(13, 145) = 2.41		
Prob > *F*	0.07			0.02			0.02			0.006		
Adjusted *R* ^2^	0.0364			0.0745			0.0730			0.1042		
Mean VIF (multicollinearity)	1.11			1.36			1.38			1.40		

*Note:* Model 1 – Parsimonious (socio‐demographic & socio‐economic) | Model 2 – + child morbidity & inflammation | Model 3—+ dietary energy & animal‐protein intake | Model 4—Final (+ hemoglobin concentration). Outcome variable: weight‐for‐age Z‐score (WAZ). Estimates are unstandardized OLS regression coefficients (*β*) with two‐tailed *p*‐values; *p < 0.05, **p < 0.01. Reference categories: Thalassemia carrier status = No; Child sex = Male. *N* = 159 for all models. Model 1 (parsimonious): Thalassemia carrier status, household asset score, household spending on food, child sex, maternal education, household food insecurity access scale score. Model 2: Model 1 + three child morbidity indicators (frequencies of loose stool, fever and cold over the past 2 weeks) + AGP (alpha‐1‐acid glycoprotein, a marker of subclinical inflammation). Model 3: Model 2 + dietary energy intake and animal‐source protein intake. Model 4 (final): Model 3 + hemoglobin concentration. Mean VIF = mean variance inflation factor across all predictors in the model.

Abbreviation: 95% CI = 95% confidence interval.

We did not report the association of thalassemia carrier and weight‐for‐height Z score (WHZ) as all four models were invalid (F statistic statistically non‐significant at *p* > 0.05).

## 5 Discussion

The study compared nutritional status and dietary intake between thalassemia non‐carrier and carrier children aged 2–5 years in Belkuchi sub‐district of Sirajganj, a north‐west district of Bangladesh. Among the key findings, thalassemia carrier children had potentially lower height‐for‐age Z‐score than their non‐carrier peers. There was no difference in the intake of macronutrients and most of the micronutrients, except for vitamins D, which the carrier children consumed lower than the non‐carriers. Household expenses on food were lower in the carrier group than its non‐carrier peers. To our knowledge, this is the first community‐based study in Bangladesh to compare both nutritional status and quantitative dietary intake between thalassemia carrier and non‐carrier young children; such evidence remains scarce globally. In contrast, Hussain et al. (2022) conducted a hospital‐based study in Pakistan focusing on body mass index (BMI) among children with thalassemia major, without assessing dietary intake. The study reported that approximately two‐thirds of the children aged 60–122 months were classified as thin or very thin, suggesting poor nutritional outcomes. Similarly, Nisha et al. (2020) reported high levels of undernutrition among thalassemic (including all three forms—minor, major, and intermedia) patients in a hospital setting in Dhaka, Bangladesh, with 33.3% severely thin and 29.1% moderately thin.

Despite the scarcity of nutritional and dietary studies among thalassemia carriers, studies are available comparing the nutritional and dietary profile in children with mild anemia vs. non‐anemic children. Mild anemia is a common feature of thalassemia carrier status (Yogalakshmi et al. 2024). A study in Indonesia among children aged 5–59 months, non‐anemic children had significantly higher intake of iron‐rich foods (liver, egg, tempeh) and much lower stunting (12% vs. 88%) than in anemic children (*p* = 0.02) (Arundito et al. 2018). Another study in Indonesia reported that anemic children had significantly higher prevalence of inadequate zinc, protein, iron, calcium, and vitamin A and B‐vitamins intake than non‐anemic children (Sunardi et al. 2021).

Unlike the hospital‐based studies in thalassemia major subjects, our study participants comprised community‐dwelling children, including thalassemia carriers (Beta thalassemia trait and hemoglobin E trait, hemoglobin D Punjab) and non‐carriers. No significant differences were observed between the carriers and non‐carriers' groups in wasting (WHZ) and underweight (WAZ), suggesting that the severe physiological impairments seen in thalassemia major are less pronounced in thalassemia carriers, thus they might be less prone for getting thin and underweight. Additionally, the study setting—an impoverished rural community—likely contributed to generally low WHZ scores among all children; the mean WHZ among non‐carriers (−0.97) was slightly below the national average (−0.93) (BDHS 2019). These combined factors may explain the lack of significant difference in wasting (WHZ) and underweight (WAZ) between the groups.

Carrier children in our study exhibited potentially lower HAZ compared to non‐carrier peers. On the contrary, the multivariable linear regression, examining the association of thalassemia carrier and HAZ showed a mixed observation. When the covariates (socio‐economics, demographics, child morbidities and dietary intakes) were added stepwise from the parsimonious to model 3, HAZ showed a consistently inverse association with thalassemia carrier, suggesting a lower growth in carriers adjusting for the pertinent and common confounders. Nonetheless, upon inclusion of hemoglobin in model 4, the association was attenuated, suggesting a tangible explanatory role of hemoglobin level to offset the preceding relationship of carrier status and linear growth of the children. Growth failure in thalassemia is predominantly reported for thalassemia major, where chronic severe anemia, ineffective erythropoiesis, iron overload, and chelation toxicity combine to impair linear growth (Arab‐Zozani et al. 2021). Severe anemia, frank ineffective erythropoiesis does not occur in the carrier/traits; thus, dyserythropoiesis related growth retardation in these children is less pronounced. Nonetheless, on a different route, the carriers are susceptible to infection (Tsai et al. 2020), subsequently requiring a higher amount of energy and key nutrients for growth and immunity, rendering them at risk of potential growth limitation. In the present study, the possible reason for the effect of lower hemoglobin in carriers to offset the inverse association of carrier status and growth is difficult to explain. As the full model (model 4) has a modest strength (Adj‐*R* ^2^ ~9.1%), the inclusion of hemoglobin might have induced statistical noise in the model to offset the association.

Further, there is a scarcity of studies assessing dietary intake in addition to nutritional status among thalassemic subjects. The study by Nisha et al. (2020) assessed Food Consumption Score (FCS) and individual‐level dietary diversity among subjects across different grades of thalassemia severity, including carriers. According to the study, around 58% of respondents reported poor or borderline food intake, while 51% and 30% reported moderate and low dietary diversity, respectively. However, the FCS assesses dietary intake at the household level and does not account for individual consumption. In contrast, the Dietary Diversity Score applies at the individual level but measures qualitative intake. A few studies have validated it, especially the Minimum Dietary Diversity for Women (MDD‐W), as an indicator of nutrient adequacy (Gómez et al. 2024; Menber et al. 2024). Nonetheless, the method remains a proxy rather than a direct measure of individual level quantitative intake.

In our study, we used a validated seven‐day semi‐quantitative Food Frequency Questionnaire (Seven‐day SQFFQ), adopted to the traditional dietary habits of Bangladeshi young children (Rahman et al. 2021). This tool enabled the estimation of actual quantitative intake, providing a better insight into comparative nutrient consumption between thalassemia carriers and non‐carriers. Our findings revealed that, among micronutrients, vitamin D intake was significantly lower among carriers than non‐carriers (*p* = 0.03), while vitamin A was borderline lower (0.07). For other micronutrients, as well as the key macronutrients and animal‐source protein, intake among carrier children was numerically lower than among non‐carriers but did not reach statistical significance. A possible explanation for the lack of statistical significance is the relatively low sample size of the thalassemia carrier group, which may have reduced the statistical power to detect differences. Despite methodological differences in dietary assessment between our study and the previously cited studies, a consistent trend emerges: thalassemia carriers generally consume lower amounts of food compared to their non‐carrier peers. This pattern is reflected in the linear growth retardation among thalassemia carriers. Furthermore, our findings of lower vitamin D intake among thalassemia carrier children support evidence linking this vitamin to linear growth in children (Bennasir et al. 2010; Ganmaa et al. 2023; Noh et al. 2019).

The mean hemoglobin concentration was lower in carriers than in non‐carriers. This is a hallmark feature of thalassemia carriers, characterized by erythroid marrow hyperplasia and ineffective erythropoiesis, which disrupts the normal tetramer structure of hemoglobin (Marengo‐Rowe 2007). A recent study conducted in a northwestern district of Bangladesh reported that the mean hemoglobin concentration was 0.4 g/dL lower among beta‐thalassemia carriers (10.5 g/dL) aged 6–37 months compared to the overall sample (10.9 g/dL) (Wendt et al. 2023).

Among other notable findings, the frequency of loose stools over the preceding 2 weeks was associated with low WAZ in models 2, 3 and 4; and with the low HAZ in the models 2 and 3. consistent with malnutrition‐infection association with reduced innate and adaptive immunity and damaged intestinal barrier (Ibrahim et al. 2017). Similar finding was observed in a large pooled analysis from 1007 children across seven cohorts in Peru, Brazil, Guinea‐Bissau, and Bangladesh and found diarrhea in the 30 days before measurement was consistently associated with lower WAZ, while cumulative diarrhea burden over time was associated with reduced length growth at 24 months (Richard et al. 2013). Household spends on food was positively associated with WAZ across all 4 models, suggesting that the economic wellbeing foster better nutrition in children. Similar observation was reported in an Indonesian study, where higher household expenditure was strongly associated with lower odds of high underweight prevalence (OR = 0.09, third vs. first tertile) (Mauludyani et al. 2014). However, a similar observation was not observed with HAZ, possibly because childhood stunting is influenced by multiple factors beyond food expenditure and intake alone. Mother's education showed a positive association with HAZ across all 4 models; and with WAZ in the models 1 and 2 (adjusted for socio‐economics, demographics and child morbidities). This may be attributed to greater awareness among educated mothers regarding child care practices, including the provision of a nutritious diet, maintenance of hygiene, and timely utilization of healthcare services (Rezaeizadeh et al. 2024).

Different types of hemoglobin disorders—Hemoglobin E Trait, Beta Thalassemia Trait, Hemoglobin E Disease, and Hemoglobin D Punjab were included in the carrier group. Hemoglobin E disease is a homozygous condition and the cases are likely to suffer more deleterious health conditions than the Traits. Despite this, there is just one case of hemoglobin E disease; inclusion of it in the carrier group might compromise generalization of health and nutritional status of the group, thus the findings need a cautious interpretation.

## 6 Limitations and Strengths

With a group size ratio of 4.5:1, the estimated statistical power to detect the group differences was 80%, which is generally considered acceptable in epidemiological studies. Nonetheless, the small group size of thalassemia carriers compared to that of non‐carriers might have undermined the statistical power to detect significant differences for most of the comparisons of nutrient intake. Further, the full regression model falling short of statistical significance to establish association of the carrier status and lower HAZ might be attributed to the low size of carriers. The residual behavior and high‐leverage data assessment provided acceptable observation; nonetheless, the possibility of unstable estimates and residual confounding in the regression models may not be ruled out. The small group size of the carriers is a definitive limitation of the study. Second, adjustment of alpha was not considered to minimize the Type 2 error, thus preventing erroneous rejection of group difference, where it is biologically plausible that the difference between the groups in nutritional intakes might exist. Nonetheless, not considering the adjustment of alpha was a limitation. Third, the study compared the nutritional and dietary profiles of thalassemia carriers and non‐carriers but did not consider other types and severity grades of thalassemia. This limits the generalizability of the findings to those groups. Fourth, the study focused on young children aged 2–5 years and did not include older age groups; thus, limiting the findings to be generalizable to the older population. Fifth, dietary intake was assessed using a validated seven‐day SQFFQ, which relies on participants' recall of the actual frequency and portion of foods consumed over the preceding 7 days (Rahman et al. 2021). This might have introduced recall bias.

Among the strengths of the study, a key advantage is the use of a quantitative dietary assessment tool, which provided better insights into nutrient intake compared to many similar studies that used qualitative methods. Second, given the unequal variances for the unequal group size, Welch's *t*‐test was used instead of the Student's *t*‐test, offering a more robust and conservative approach that protects from inflation of the risk of Type 1 error (Delacre et al. 2017). Third, simple random sampling was used to select the non‐carrier children from its pool, enhancing the representativeness of the sample.

## 7 Conclusion

In conclusion, among children aged 2–5 years residing in rural Bangladesh, the intake of most macro‐ and micronutrients did not differ between the thalassemia carrier and the non‐thalassemic peers, except that the carriers consumed lower amounts of vitamin D, the nutrient essential for growth. Mother's education favored child growth and was protective of underweight. Higher spending on food was protective of underweight. A higher frequency of loose stools was observed in children with underweight and, to some extent, in length‐faltering children. Thalassemic carrier children had lower length growth potential compared to non‐thalassemic peers. Taking into account the pertinent explanatory factors—socio‐demographics, child morbidities, dietary intakes, and hemoglobin concentration, overall, the thalassemia carrier state was not associated with low length growth in young children. Larger research is required for additional insights on the comparative nutritional profile between children with thalassemia carriers and non‐carriers.

## Author Contributions

**Sabuktagin Rahman:** conceptualization, methodology, data curation, investigation, formal analysis, supervision, project administration, writing – original draft, writing – review and editing. **Farhana Rinky:** conceptualization, methodology, formal analysis, writing – original draft. **Santhia Ireen:** conceptualization, methodology, formal analysis, writing – review and editing. **Faruk Ahmed:** conceptualization, methodology, validation, supervision, funding acquisition, writing – review and editing.

## Funding

The research was funded by the Nestlé Foundation.

## Consent

Written informed consents were obtained from all participants.

## Conflicts of Interest

The authors declare no conflicts of interest.

## Data Availability

The datasets used and analyzed during the current study are available from the corresponding author upon reasonable request.

## References

[fsn372545-bib-0001] Ara, U. N. , T. Chowdhury , S. Akhter , T. Sultana , and M. A. Torsa . 2023. “Growth Parameters in Children With Transfusion‐Dependent Thalassemia and Their Correlation With Serum Ferritin.” Dhaka Shishu (Children) Hospital Journal 39, no. 2: 88–92. 10.3329/dshj.v39i2.82856.

[fsn372545-bib-0002] Arab‐Zozani, M. , S. Kheyrandish , A. Rastgar , and E. Miri‐Moghadam . 2021. “A Systematic Review and Meta‐Analysis of Stature Growth Complications in β‐Thalassemia Major Patients.” Annals of Global Health 87, no. 1: 48. 10.5334/aogh.3184. PMC8194969 34164261

[fsn372545-bib-0003] Arini, D. , N. Nursalam , M. Mahmudah , and I. Faradilah . 2020. “The Incidence of Stunting, the Frequency/Duration of Diarrhea and Acute Respiratory Infection in Toddlers.” Journal of Public Health Research 9, no. 2: 1816. 10.4081/jphr.2020.1816. PMC7376488 32728562

[fsn372545-bib-0004] Arundito, B. W. , A. Senolinggi , and E. P. Prawirohartono . 2018. “Comparison of Dietary Sources of Iron, Nutritional Status and Family Factors Between Anemic and Non‐Anemic Children in Sorong, West Papua Province, Indonesia.” Asia Pacific Journal of Pediatrics and Child Health 2, no. 4: 25–40. https://apjpch.com/pdfs/181284bA172134.pdf.

[fsn372545-bib-0005] Ayukarningsih, Y. , J. Amalia , and G. Nurfarhah . 2022. “Thalassemia and Nutritional Status in Children.” Journal of Health and Dental Sciences 2, no. 1: 39–52.

[fsn372545-bib-0006] Barik, S. , D. A. Nagdeve , A. Singh , and M. Singh . 2026. “Son Preference and Regional Inequalities in Girls Undernutrition in India.” Scientific Reports 16: 22620. 10.1038/s41598-026-52420-y. PMC13381677 42156457

[fsn372545-bib-0007] BBS . 2024. “National Thalassemia Survey‐2024 Key Findings Electrophoresis Test Results.” https://file‐dhaka.portal.gov.bd/uploads/3697a1e4‐b2b6‐4d06‐9f49‐963e915ea58c//66c/6be/a85/66c6bea8543f1751997110.pdf.

[fsn372545-bib-0008] BDHS . 2019. Bangladesh Demographic and Health Survey 2017–18. DHS Program.

[fsn372545-bib-0009] BDHS . 2022. “Demographic and Health Survey.” https://dhsprogram.com/pubs/pdf/FR386/FR386.pdf.

[fsn372545-bib-0010] Bennasir, H. , S. Sridhar , and T. T. Abdel‐Razek . 2010. “Vitamin A From Physiology to Disease Prevention.” International Journal of Pharmaceutical Sciences Review and Research 1, no. 1: 68–73.

[fsn372545-bib-0011] Biswas, B. , N. N. Naskar , K. Basu , A. Dasgupta , R. Basu , and B. Paul . 2020. “Malnutrition, Its Attributes, and Impact on Quality of Life: An Epidemiological Study Among β‐Thalassemia Major Children.” Korean Journal of Family Medicine 42, no. 1: 66–72. 10.4082/kjfm.19.0066. PMC7884889 31955549

[fsn372545-bib-0012] Cashin, K. , and L. Oot . 2018. “Guide to Anthropometry: A Practical Tool for Program Planners, Managers, and Implementers.” Food Nutr Tech Assist III Proj (FANTA)/FHI 360: 1–231.

[fsn372545-bib-0013] Coates, J. , A. Swindale , and P. Bilinsky . 2007. Household Food Insecurity Access Scale (HFIAS) for Measurement of Food Access: Indicator. Food and Nutrition Technical Assistance III Project (FANTA). https://www.fantaproject.org/sites/default/files/resources/HFIAS_ENG_v3_Aug07.pdf.

[fsn372545-bib-0014] Correia, M. I. T. D. 2018. “Addressing the Hidden Burden of Malnutrition for Hospitalized Patients.” Journal of the Academy of Nutrition and Dietetics 118, no. 1: 37–39. 10.1016/j.jand.2017.03.009. 28416433

[fsn372545-bib-0015] Delacre, M. , D. Lakens , and C. Leys . 2017. “Why Psychologists Should by Default Use Welch's t‐Test Instead of Student's t‐Test.” International Review of Social Psychology 30, no. 1: 92–101. 10.5334/irsp.82.

[fsn372545-bib-0016] Eleftheriou, A. , and M. Angastiniotis . 2021. Global Thalassaemia Review 2021. Thalassaemia International Federation. 41564232

[fsn372545-bib-0017] Faizan, M. , N. Rashid , S. Hussain , et al. 2023. “Prevalence and Clinical Features of Thalassemia Minor Cases.” Pakistan Journal of Medical and Health Sciences 17, no. 2: 456–459.

[fsn372545-bib-0018] Fung, E. B. 2010. “Nutritional Deficiencies in Patients With Thalassemia.” Annals of the New York Academy of Sciences 1202, no. 1: 188–196. 10.1111/j.1749-6632.2010.05578.x. 20712792

[fsn372545-bib-0019] Galanello, R. , and R. Origa . 2010. “Beta‐Thalassemia.” Orphanet Journal of Rare Diseases 5, no. 1: 11. 10.1186/1750-1172-5-11. PMC2893117 20492708

[fsn372545-bib-0020] Ganmaa, D. , S. Bromage , P. Khudyakov , S. Erdenenbaatar , B. Delgererekh , and A. R. Martineau . 2023. “Influence of Vitamin D Supplementation on Growth, Body Composition, and Pubertal Development Among School‐Aged Children in an Area With a High Prevalence of Vitamin D Deficiency: A Randomized Clinical Trial.” JAMA Pediatrics 177, no. 1: 32–41. 10.1001/jamapediatrics.2022.4581. PMC9706398 36441522

[fsn372545-bib-0021] Goldberg, E. K. , A. Lal , and E. B. Fung . 2022. “Nutrition in Thalassemia: A Systematic Review of Deficiency, Relations to Morbidity, and Supplementation Recommendations.” Journal of Pediatric Hematology/Oncology 44, no. 1: 1–11. 10.1097/MPH.0000000000002291. PMC8732300 34486568

[fsn372545-bib-0022] Gómez, G. , R. Monge‐Rojas , R. Vargas‐Quesada , et al. 2024. “Exploring the FAO Minimum Dietary Diversity Indicator as a Suitable Proxy of Micronutrient Adequacy in Men and Women Across Reproductive and Non‐Reproductive Ages in 8 Latin American Countries.” Food and Nutrition Bulletin 45, no. 2_suppl: S55–S65. 10.1177/03795721241242920. 38807356

[fsn372545-bib-0023] Guimaraes, J. S. , J. G. Cominal , A. C. Silva‐Pinto , et al. 2015. “Altered Erythropoiesis and Iron Metabolism in Carriers of Thalassemia.” European Journal of Haematology 94, no. 6: 511–518. 10.1111/ejh.12464. PMC4393762 25307880

[fsn372545-bib-0024] Headey, D. , K. Hirvonen , and J. Hoddinott . 2018. “Animal Sourced Foods and Child Stunting.” American Journal of Agricultural Economics 100, no. 5: 1302–1319. 10.1093/ajae/aay053. PMC7734193 33343003

[fsn372545-bib-0025] Hossain, M. S. , F. Islam , S. Akhter , and A. Al Mossabbir . 2025. “Thalassemia in Bangladesh: Progress, Challenges, and a Strategic Blueprint for Prevention.” Orphanet Journal of Rare Diseases 20, no. 1: 358. 10.1186/s13023-025-03744-x. PMC12247199 40640823

[fsn372545-bib-0026] Hussain, A. , M. Qasim Khan , K. Ahmad , H. U. Rahman , M. Fazil , and M. Qasim . 2022. “Evaluation of Children's Nutritional Status With Beta Thalassemia Major Based on BMI.” Journal of Pharmaceutical Negative Results 13, no. 4: 2205–2209. 10.47750/pnr.2022.13.04.297.

[fsn372545-bib-0027] Ibrahim, M. K. , M. Zambruni , C. L. Melby , and P. C. Melby . 2017. “Impact of Childhood Malnutrition on Host Defense and Infection.” Clinical Microbiology Reviews 30, no. 4: 919–971. 10.1128/CMR.00119-16. PMC5608884 28768707

[fsn372545-bib-0028] Jawed, I. , M. O. Alam , F. B. Fatima , S. A. Johar , and S. A. M. Shah . 2024. “Case Report—Unveiling a Case of Hemoglobin D‐Punjab Variant With Iron Deficiency Anemia in Sindh (Pakistan).” SAGE Open Medical Case Reports 12: 2050313X241272516. 10.1177/2050313X241272516. PMC11331452 39161918

[fsn372545-bib-0029] Mahfouz, E. M. , E. S. Mohammed , S. F. Alkilany , and T. A. A. Rahman . 2022. “The Relationship Between Dietary Intake and Stunting Among Pre‐School Children in Upper Egypt.” Public Health Nutrition 25, no. 8: 2179–2187. 10.1017/s136898002100389x. PMC9991819 34496999

[fsn372545-bib-0030] Marengo‐Rowe, A. J. 2007. “The Thalassemias and Related Disorders.” Baylor University Medical Center Proceedings 20, no. 1: 27–31. 10.1080/08998280.2007.11928230. PMC1769530 17256039

[fsn372545-bib-0031] Mashon, R. S. , K. Mandrelle , and N. Kakkar . 2025. “Experience of Antenatal Thalassemia Screening and Prenatal Diagnosis From a Tertiary Care Teaching Hospital in Punjab.” Indian Journal of Hematology and Blood Transfusion 41, no. 1: 96–101. 10.1007/s12288-024-01785-z. PMC11794726 39917488

[fsn372545-bib-0032] Mauludyani, A. V. R. , U. Fahmida , and O. Santika . 2014. “Relationship Between Household Expenditures on Strategic Foods and Prevalence of Undernutrition Among Children 0 to 23 Months of Age in Indonesia.” Food and Nutrition Bulletin 35, no. 4: 440–448. 10.1177/156482651403500406. 25639129

[fsn372545-bib-0033] Menber, Y. , S. Gashaw , T. Belachew , and N. Fentahun . 2024. “Validation of the Minimum Dietary Diversity for Women as a Predictor of Micronutrient Adequacy Among Lactating Women in Ethiopia.” Frontiers in Nutrition 11: 1459041. 10.3389/fnut.2024.1459041. PMC11446886 39364155

[fsn372545-bib-0034] Mirhosseini, N. Z. , S. Shahar , M. Ghayour‐Mobarhan , et al. 2012. “Factors Affecting Nutritional Status Among Pediatric Patients With Transfusion‐Dependent Beta Thalassemia.” Mediterranean Journal of Nutrition and Metabolism 6, no. 1: 45–51. 10.3233/s12349-012-0112-0.

[fsn372545-bib-0035] Musallam, K. M. , L. Lombard , K. D. Kistler , et al. 2023. “Epidemiology of Clinically Significant Forms of Alpha‐and Beta‐Thalassemia: A Global Map of Evidence and Gaps.” American Journal of Hematology 98, no. 9: 1436–1451. 10.1002/ajh.27006. 37357829

[fsn372545-bib-0036] Musallam, K. M. , A. T. Taher , M. D. Cappellini , and V. G. Sankaran . 2013. “Clinical Experience With Fetal Hemoglobin Induction Therapy in Patients With β‐Thalassemia.” Blood 121, no. 12: 2199–2212. 10.1182/blood-2012-10-408021. 23315167

[fsn372545-bib-0038] Mustafa, M. , A. Thiru , E. M. Illzam , et al. 2016. “Pathophysiology, Clinical Manifestations, and Carrier Detection in Thalassemia.” IOSR Journal of Dental and Medical Sciences 15, no. 11: 122–126.

[fsn372545-bib-0037] Na, M. , A. A. Shamim , S. Mehra , et al. 2020. “Maternal Nutritional Status Mediates the Linkage Between Household Food Insecurity and Mid‐Infancy Size in Rural Bangladesh.” British Journal of Nutrition 123, no. 12: 1415–1425. 10.1017/S0007114520000707. 32102702

[fsn372545-bib-0039] Ndovie, P. , S. G. Nkhata , N. Geresomo , R. Fungo , V. Nyau , and J. Munthali . 2025. “Socio‐Economic and Demographic Determinants of Undernutrition Among 6–59 Months Old Children Living in Malawian Stunting Hotspots: A Cross‐Sectional Community Study.” BMC Nutrition 11, no. 1: 14. 10.1186/s40795-025-01001-9. PMC11745028 39833945

[fsn372545-bib-0040] Nisha, S. , S. S. Alam , M. N. Rahman , and K. Islam . 2020. “Nutritional Status and Dietary Patterns of Thalassemia Patients at Selected Hospitals in Dhaka City, Bangladesh.” Novel Techniques in Nutrition and Food Science 5, no. 2: 431–434.

[fsn372545-bib-0041] Noh, M. F. M. , R. D. N. Gunasegavan , and S. Mustar . 2019. “Vitamin A in Health and Disease.” In Vitamin A, edited by L. Q. Zepka , V. V. de Rosso , and E. Jacob‐Lopes . IntechOpen. 10.5772/intechopen.84460.

[fsn372545-bib-0042] Noor, F. A. , N. Sultana , G. S. Bhuyan , et al. 2020. “Nationwide Carrier Detection and Molecular Characterization of β‐Thalassemia and Hemoglobin E Variants in Bangladeshi Population.” Orphanet Journal of Rare Diseases 15, no. 1: 15. 10.1186/s13023-020-1294-z. PMC6961315 31941534

[fsn372545-bib-0043] Nyirenda, N. , H. Muturi , and C. Muyuni . 2025. “Higher Maternal Education Is Associated With Reduced Childhood Stunting in Zambia: Findings From the 2018 DHS.” *MedRxiv*. 10.1101/2025.06.10.25329376.

[fsn372545-bib-0044] Perneger, T. V. 1998. “What's Wrong With Bonferroni Adjustments.” BMJ 316, no. 7139: 1236–1238. 10.1136/bmj.316.7139.1236. PMC1112991 9553006

[fsn372545-bib-0045] Peters, M. , H. Heijboer , F. Smiers , and P. C. Giordano . 2012. “Diagnosis and Management of Thalassaemia.” BMJ 344: e228. 10.1136/bmj.e228. 22277544

[fsn372545-bib-0046] Rahayuwati, L. , M. Komariah , C. W. M. Sari , et al. 2023. “The Influence of Mother's Employment, Family Income, and Expenditure on Stunting Among Children Under Five: A Cross‐Sectional Study in Indonesia.” Journal of Multidisciplinary Healthcare 16: 2271–2278. 10.2147/JMDH.S417749. PMC10437103 37601326

[fsn372545-bib-0047] Rahman, S. , P. Lee , S. Ireen , and F. Ahmed . 2021. “Validation of an Interviewer‐Administered Seven‐Day Semi‐Quantitative Food Frequency Questionnaire for the Dietary Assessment of Preschool Children in Rural Bangladesh.” Journal of Nutritional Science 10: e26. 10.1017/jns.2021.19. PMC8080233 33996039

[fsn372545-bib-0048] Rahman, S. , P. Lee , M. R. Khan , and F. Ahmed . 2023. “Thalassemia Carrier Status and Groundwater Iron: Implication for Iron Supplementation Program for Children in Bangladesh.” Journal of Public Health Research 12, no. 3: 22799036231191036. 10.1177/22799036231191036. PMC10439722 37602276

[fsn372545-bib-0049] Rahman, S. , P. Lee , R. Raqib , A. K. Roy , M. R. Khan , and F. Ahmed . 2019. “Effect of Micronutrient Powder (MNP) With a Low‐Dose of Iron on Hemoglobin and Iron Biomarkers, and Its Effect on Morbidities in Rural Bangladeshi Children Drinking Groundwater With a High‐Level of Iron: A Randomized Controlled Trial.” Nutrients 11, no. 11: 2756. 10.3390/nu11112756. PMC6893643 31766250

[fsn372545-bib-0050] Random.org . 2026. “RANDOM.ORG—True Random Number Service.” https://www.random.org/.

[fsn372545-bib-0051] Rezaeizadeh, G. , M. A. Mansournia , A. Keshtkar , et al. 2024. “Maternal Education and Its Influence on Child Growth and Nutritional Status During the First Two Years of Life: A Systematic Review and Meta‐Analysis.” EClinicalMedicine 71: 102574. 10.1016/j.eclinm.2024.102574. PMC11001623 38596614

[fsn372545-bib-0052] Richard, S. A. , R. E. Black , R. H. Gilman , et al. 2013. “Diarrhea in Early Childhood: Short‐Term Association With Weight and Long‐Term Association With Length.” American Journal of Epidemiology 178, no. 7: 1129–1138. 10.1093/aje/kwt094 PMC3783094 23966558

[fsn372545-bib-0053] Sadiq, I. Z. , F. S. Abubakar , H. S. Usman , et al. 2024. “Thalassemia: Pathophysiology, Diagnosis, and Advances in Treatment.” Thalassemia Reports 14, no. 4: 81–102. 10.3390/thalassrep14040010.

[fsn372545-bib-0054] Schlaudecker, E. P. , M. C. Steinhoff , and S. R. Moore . 2011. “Interactions of Diarrhea, Pneumonia, and Malnutrition in Childhood: Recent Evidence From Developing Countries.” Current Opinion in Infectious Diseases 24, no. 5: 496–502. 10.1097/QCO.0b013e328349287d PMC5454480 21734569

[fsn372545-bib-0055] Schnedl, W. J. , M. Schenk , S. Lackner , S. J. Holasek , and H. Mangge . 2017. “β‐Thalassemia Minor, Carbohydrate Malabsorption and Histamine Intolerance.” Journal of Community Hospital Internal Medicine Perspectives 7, no. 4: 227–229. 10.1080/20009666.2017.1369378. PMC5637652 29046748

[fsn372545-bib-0056] Semba, R. D. , S. de Pee , K. Sun , M. Sari , N. Akhter , and M. W. Bloem . 2008. “Effect of Parental Formal Education on Risk of Child Stunting in Indonesia and Bangladesh: A Cross‐Sectional Study.” Lancet 371, no. 9609: 322–328. 10.1016/S0140-6736(08)60169-5. 18294999

[fsn372545-bib-0057] Shaheen, N. , L. Bari , and M. A. Mannan . 2013. Food Composition Table for Bangladesh. University of Dhaka.

[fsn372545-bib-0058] Shang, X. , and X. Xu . 2017. “Update in the Genetics of Thalassemia: What Clinicians Need to Know.” Best Practice & Research. Clinical Obstetrics & Gynaecology 39: 3–15. 10.1016/j.bpobgyn.2016.10.012. 27876354

[fsn372545-bib-0059] Skordis, N. , and A. Kyriakou . 2011. “The Multifactorial Origin of Growth Failure in Thalassaemia.” Pediatric Endocrinology Reviews: PER 8: 271–277. 21705977

[fsn372545-bib-0060] Soliman, A. , M. Yassin , F. Alyafei , et al. 2023. “Nutritional Studies in Patients With β‐Thalassemia Major: A Short Review.” Acta Bio Medica: Atenei Parmensis 94, no. 3: e2023187. 10.23750/abm.v94i3.14732. PMC10308461 37326258

[fsn372545-bib-0061] StatsCalculators . 2026. “StatsCalculators.com—Free Online Statistics Calculators.” https://www.statscalculators.com/.

[fsn372545-bib-0062] Suh, J. R. , A. K. Herbig , and P. J. Stover . 2001. “New Perspectives on Folate Catabolism.” Annual Review of Nutrition 21, no. 1: 255–282. 10.1146/annurev.nutr.21.1.255. 11375437

[fsn372545-bib-0063] Sunardi, D. , S. Bardosono , R. W. Basrowi , E. Wasito , and Y. Vandenplas . 2021. “Dietary Determinants of Anemia in Children Aged 6–36 Months: A Cross‐Sectional Study in Indonesia.” Nutrients 13, no. 7: 2397. 10.3390/nu13072397. PMC8308564 34371908

[fsn372545-bib-0064] Suresh, H. , S. Jamil , B. K. Padhi , and M. J. Hossain . 2023. “Thalassemia Prevention: Religious and Cultural Barriers to Premarital Screening in Bangladesh.” Health Science Reports 6, no. 4: e1176. 10.1002/hsr2.1176. PMC10075244 37033390

[fsn372545-bib-0065] Thurnham, D. I. , C. A. Northrop‐Clewes , and J. Knowles . 2015. “The Use of Adjustment Factors to Address the Impact of Inflammation on Vitamin A and Iron Status in Humans.” Journal of Nutrition 145, no. 5: 1137S–1143S. 10.3945/jn.114.194712. PMC4410494 25833890

[fsn372545-bib-0066] Tsai, T.‐A. , C.‐K. Tsai , Y.‐H. Yang , et al. 2020. “Higher Hospitalization Rate for Lower Airway Infection in Transfusion‐naïve Thalassemia Children.” Frontiers in Pediatrics 8: 574014. 10.3389/fped.2020.574014. PMC7732513 33330275

[fsn372545-bib-0067] USDA . 2026. “Food Search | USDA FoodData Central.” https://fdc.nal.usda.gov/food‐search.

[fsn372545-bib-0068] van Neerven, R. J. J. 2025. “Macronutrients, Micronutrients, and Malnutrition: Effects of Nutrition on Immune Function in Infants and Young Children.” Nutrients 17, no. 9: 1469. 10.3390/nu17091469 PMC12073586 40362777

[fsn372545-bib-0069] Vongsakit, S. , K. Ohara , Y. Fujita , A. Takada , and K. Kouda . 2025. “Household Wealth Index Is Associated With Stunting Among Children Under 5: A Cross‐Sectional Analysis of the Lao Social Indicator Survey II.” Journal of Physiological Anthropology 44, no. 1: 20. 10.1186/s40101-025-00402-w. PMC12254960 40652261

[fsn372545-bib-0070] Vyas, S. , and L. Kumaranayake . 2006. “Constructing Socio‐Economic Status Indices: How to Use Principal Components Analysis.” Health Policy and Planning 21, no. 6: 459–468. 10.1093/heapol/czl029. 17030551

[fsn372545-bib-0071] Wang, L. , L. Zhang , Y. Yang , Y. Luo , L. Wang , and S. Patil . 2024. “Association of Age to Nutritional Status and Muscle Mass in Children With Transfusion‐Dependent β‐Thalassemia: A Cross‐Sectional Study.” Frontiers in Nutrition 11: 1493502. 10.3389/fnut.2024.1493502. PMC11543532 39525502

[fsn372545-bib-0072] Weisz, A. , G. Meuli , C. Thakwalakwa , I. Trehan , K. Maleta , and M. Manary . 2011. “The Duration of Diarrhea and Fever Is Associated With Growth Faltering in Rural Malawian Children Aged 6‐18 Months.” Nutrition Journal 10, no. 1: 25. 10.1186/1475-2891-10-25 PMC3068082 21418600

[fsn372545-bib-0073] Wendt, A. S. , J. Brintrup , J. L. Waid , A. Kader , N. J. Lambrecht , and S. Gabrysch . 2023. “Thalassemia and Hemoglobinopathy Prevalence in a Community‐Based Sample in Sylhet, Bangladesh.” Orphanet Journal of Rare Diseases 18, no. 1: 192. 10.1186/s13023-023-02821-3 PMC10355052 37468973

[fsn372545-bib-0074] WHO . 2006. WHO Child Growth Standards: Length/Height‐For‐Age, Weight‐For‐Age, Weight‐For‐Length, Weight‐For‐Height and Body Mass Index‐For‐Age: Methods and Development. World Health Organization.

[fsn372545-bib-0075] Yogalakshmi, E. , S. Vasudevan , S. Sonti , K. Kannan , and C. Srinivasan . 2024. “Exploring the Clinical and Hematological Characteristics of Beta‐Thalassemia Trait: A Comprehensive Analysis in a Tertiary Care Hospital Setting.” Cureus 16, no. 5: e61093. 10.7759/cureus.61093 PMC11198867 38919232

[fsn372545-bib-0076] Zaharia, S. , S. Ghosh , R. Shrestha , et al. 2021. “Sustained Intake of Animal‐Sourced Foods Is Associated With Less Stunting in Young Children.” Nature Food 2, no. 4: 246–254. 10.1038/s43016-021-00259-z. 37118465

